# Rapid recovery following short-term acoustic disturbance in two fish species

**DOI:** 10.1098/rsos.150686

**Published:** 2016-01-27

**Authors:** Rick Bruintjes, Julia Purser, Kirsty A. Everley, Stephanie Mangan, Stephen D. Simpson, Andrew N. Radford

**Affiliations:** 1 School of Biological Sciences, University of Bristol, Bristol BS8 1TQ, UK; 2 Biosciences, College of Life and Environmental Sciences, University of Exeter, Exeter EX4 4QD, UK; 3 HR Wallingford, Howbery Park, Wallingford OX10 8BA, UK

**Keywords:** anthropogenic noise, behaviour, residual effect, physiology, environmental pollutant, sound

## Abstract

Noise from human activities is known to impact organisms in a variety of taxa, but most experimental studies on the behavioural effects of noise have focused on examining responses associated with the period of actual exposure. Unlike most pollutants, acoustic noise is generally short-lived, usually dissipating quickly after the source is turned off or leaves the area. In a series of experiments, we use established experimental paradigms to examine how fish behaviour and physiology are affected, both during short-term (2 min) exposure to playback of recordings of anthropogenic noise sources and in the immediate aftermath of noise exposure. We considered the anti-predator response and ventilation rate of juvenile European eels (*Anguilla anguilla*) and ventilation rate of juvenile European seabass (*Dicentrarchus labrax*). As previously found, additional-noise exposure decreased eel anti-predator responses, increased startle latency and increased ventilation rate relative to ambient-noise-exposed controls. Our results show for the first time that those effects quickly dissipated; eels showed rapid recovery of startle responses and startle latency, and rapid albeit incomplete recovery of ventilation rate in the 2 min after noise cessation. Seabass in both laboratory and open-water conditions showed an increased ventilation rate during playback of additional noise compared with ambient conditions. However, within 2 min of noise cessation, ventilation rate showed complete recovery to levels equivalent to ambient-exposed control individuals. Care should be taken in generalizing these rapid-recovery results, as individuals might have accrued other costs during noise exposure and other species might show different recovery times. Nonetheless, our results from two different fish species provide tentative cause for optimism with respect to recovery following short-duration noise exposure, and suggest that considering periods following noise exposures could be important for mitigation and management decisions.

## 1. Introduction

Most organisms will endure some degree of anthropogenic disturbance, such as global warming, ocean acidification or chemical pollution, during their lives. These disturbances, and their impacts, are predicted to persist for some time even after discontinuation of the underlying cause [1–3]. Anthropogenic noise, a pollutant of international concern (appearing in, for example, the US National Environmental Policy Act and the European Commission Marine Strategy Framework Directive), potentially differs in this regard. While there is often a background level of distant anthropogenic noise (e.g. from shipping), as well as constant sound arising from abiotic and biotic sources, once a particular anthropogenic noise source is silenced or moves away, almost no lasting physical trace of its sound remains.

Increasing amounts of research have provided evidence that several sources of anthropogenic noise can affect at least some species in a range of taxa, including invertebrates, fishes, birds and mammals [4–7]. Exposure to additional noise can have subtle effects and has, for example, been found to impact individual and group behaviour [8,9], but also to compromise community structure [10,11]. More severe impacts of noise, such as permanent hearing damage, lesions or death [12–16], are expected in cases of high receiver hearing sensitivity, prolonged duration of noise exposure and high noise intensity (e.g. [17,18]). In fishes, direct impacts of noise have been shown to cause stress [19,20] and injuries [12,21], or affect catch rates [22], communication [23], feeding rate [24,25], anti-predator behaviour [26,27] and swimming [28,29]. Moreover, direct responses can vary depending on, for example, context, prior experience or body condition [8,30,31].

Several effects of noise in fishes have been shown to persist following noise exposure during background noise levels. These post-exposure effects include high stress levels [19], increased cardiac output [32], reduced catch rates [22] and temporary hearing loss [33–36]. Other potential lasting impacts of noise on physiology or behaviour have rarely been explored (but see [29,37]). This is important, as considering whether there are residual effects of noise or rapid recovery following noise exposure, in the absence of physiological injury, could be essential for mitigation and management strategies. Additionally, while highly intensive noise sources, for example pile driving in shallow water, tend to be regional in their scale of impact [38], most organisms will typically be exposed to less intense anthropogenic noise levels.

Recent laboratory-based experiments have demonstrated that juvenile European eels (*Anguilla anguilla*) are less likely to startle to a looming predatory stimulus and exhibit increased ventilation rates (indicative of greater stress) during exposure to additional noise (playback of recordings of ships passing through harbours) compared to control conditions (playback of recordings of the same harbours without ships) [26]. Using these established methods, we carried out new experiments to examine whether juvenile eels continue to exhibit behavioural and physiological responses directly after exposure to a single short-term noise-pollution event ceases, or whether they show rapid recovery in the aftermath. We also used the ventilation-rate experimental paradigm to consider the effect of a different additional noise (playback of pile driving) on a second species (European seabass, *Dicentrachus labrax*). Experiments in both laboratory and open-water conditions were used to test whether additional noise had an effect and if that effect was sustained once the noise ceased.

## 2. Material and methods

### 2.1 Eels

Glass-stage juvenile European eels, collected from the River Severn and weaned by Glass Eels Ltd (Gloucestershire, UK), were held in 450 l glass aggregation tanks at the University of Bristol. Minimally, one week prior to experiments, fish were transferred to 50 l glass holding tanks located in experimental rooms. Light:dark regimes were 13 L:11 D with constant temperature (16±0.2°C) and the fish were fed once daily (Perle eel food, Skretting, Norway). European eels can perceive sound pressure frequencies lower than 300 Hz [39], coinciding with the dominant frequencies of ship noise [40].

### 2.2 Eel sound recordings and playbacks

**Figure 1.**
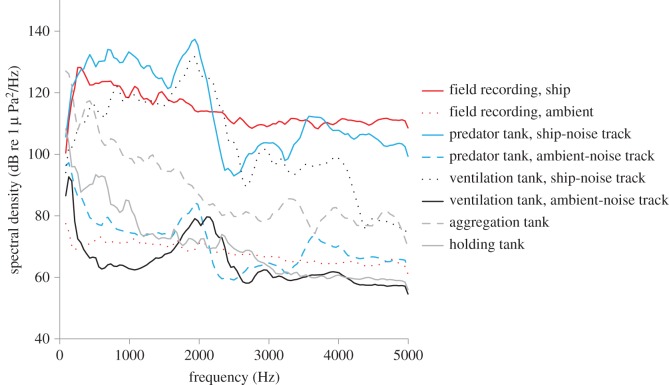
Spectral-level analyses of field and tank-based recordings for eels. The figure includes baseline conditions in the aggregation and holding tanks, field recordings of ambient harbour noise and ship noise (recorded in Plymouth harbour), and ambient-noise and additional-noise playback in each type of test tank; for anti-predator experiments (Predator tank) and ventilation rate experiments (Ventilation tank). Fast Fourier Transform (FFT) analysis of sound 50–5000 Hz, using Avisoft SASLabPro v. 5.2.07 (Avisoft Bioacoustics): spectrum-level units normalized to 1 Hz bandwidth, Hann evaluation window, 50% overlap, FFT size 1024, averaged from a 15 s sample of each recording, 43 Hz intervals are presented.

Sound recordings of baseline captive noise conditions were made 2 cm above the tank bottom at the end of the aggregation tanks—where the eels usually rested—and in the centre of the smaller holding tanks (see figure 1 for acoustic conditions). In the predation experiment, sound recordings were made 2 cm above the tank bottom in the middle of the test tank; in the ventilation experiment, recordings were made 10 cm from the speaker just above the tank floor (where the individuals were tested). All sound recordings were made using a Hi Tech Inc. HTI-96-MIN omnidirectional hydrophone (with inbuilt preamplifier, manufacturer calibrated sensitivity −164.3 dB re 1 V/μPa, 0.02–30 kHz frequency range) and a Roland Edirol recorder (R09HR 24-bit, 44.1 kHz sampling rate, calibrated using a pure sine wave reference signal of known amplitude and measured on an oscilloscope). The reference signal was produced by a function generator (TTi RS Components 216-069, TG230, 2 MHz sweep/function generator). Due to unresolved challenges in measuring particle motion in small tanks and in the near field, acoustic conditions were assessed in the pressure domain only (see [41] for detailed considerations of complex pressure and particle motion conditions in tanks). Although eels (and seabass; see later) are sensitive to particle motion as well as pressure [39,42], absolute values for sensitivity were not established. We rather explored the direct impacts of additional noise on physiology and behaviour, as well as its impact immediately post-exposure.

**Figure 2.**
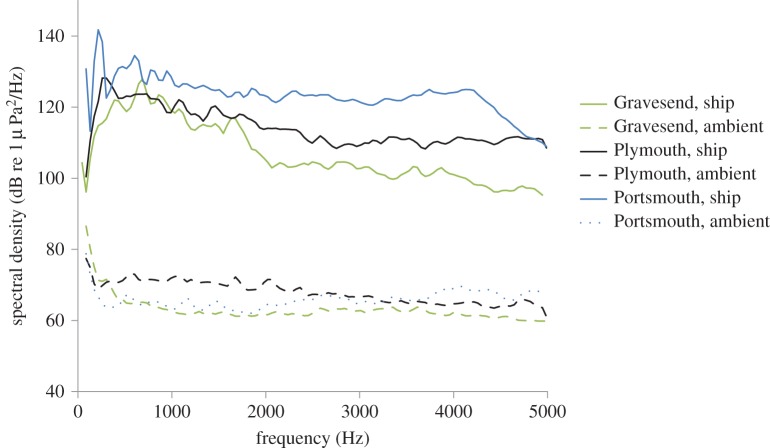
Spectral-level analyses of field recordings made in three different harbours (Gravesend, Plymouth and Portsmouth, UK) during ambient harbour noise and the passing of a ship. FFT analysis of sound 50–5000 Hz, using Avisoft SASLabPro v. 5.2.07: spectrum-level units normalized to 1 Hz bandwidth, Hann evaluation window, 50% overlap, FFT size 1024, averaged from a 15 s sample of each recording, 43 Hz intervals are presented.

Original recordings of both ambient noise and the noise of slowly passing (less than 10 knots) commercial ships in the same location were made at three large UK harbours at *ca* 100–400 m distance (Portsmouth Commodore Goodwill, ferry, 126 m length, 5215 tons; Plymouth: Bro Distributor, LPG tanker, 147 m length, 14 500 tons; Gravesend: Rio de la Plata, container ship, 286 m length, 64 730 tons; see figure 2 for individual recordings). Acoustic differences between the recordings can be due to, for example, the different primary noise sources, tides, currents, waves, water depth and distant anthropogenic noise sources. Recordings were cut into 2-min segments using Audacity 1.3.13 (http://audacity.sourceforge.net/) to create three different ambient-noise and ship-noise playback tracks (one each from each harbour). A period of 2 min was chosen as this was close to the time it took for ships to pass the harbours where the recordings were made. As recommended in numerous papers (e.g. [43,44]), and as is standard good experimental practice, multiple playback tracks of each treatment type were used to reduce pseudo-replication, and thus allow generalization of results; this methodology ensures that potential behavioural and physiological responses were not specific to one particular additional-noise or ambient playback track. Sound levels of the tracks of each type were modified to play at approximately equal pressure levels, when played back using a UW-30 underwater speaker (frequency range 0.1–10 kHz; Lubell Labs Inc., Columbus, OH, USA), an M033N amplifier (Kemo Electronics GmbH, Langen, Germany; 18 W; frequency range *ca* 0.04–20 kHz) powered by a battery (12 V, 7.2 Ah sealed lead-acid), with a Logic L2GMP309 player (frequency response range: 40–20 000 Hz). The three ambient-noise playbacks in the centre of the predation tank (see below) had received levels between 105.3 and 107.6 dB RMS re 1 μPa, whereas the ship-noise playbacks had received levels between 144.4 and 148.06 dB RMS re 1 μPa. In the ventilation tank (see below), ambient-noise playbacks in the location where the fish were tested measured between 105.1 and 108.6 dB RMS re 1 μPa, while ship-noise playbacks measured between 139.3 and 141.2 dB RMS re 1 μPa; all RMS values were calculated over 1 min, frequency range 50–5000 Hz.

### 2.3 Eel predation experiment

We examined potential immediate and lasting impacts of additional noise on eel anti-predator behaviour using a looming-stimulus methodology (e.g. [26,45,46]), which simulates an ambush predatory threat. A model ‘predator fish’ (a red wooden simulated fish head; size 12×10 cm) on a 45° swinging pendulum arm was set up outside one short side of the experimental tank; the model moved to a position next to, but not touching, the exterior tank-wall upon release. The experiment was conducted in a glass tank (size 120×40 cm; wall thickness: 4 mm) with a 40 cm water depth. An upward-facing underwater loudspeaker was situated under a false bottom made of Perspex (thickness: 4 mm), and the experimental tank and the observer were separated by a blind.

For each trial, a new eel was caught from the holding tank, transferred in a translucent jug and released into the experimental tank at the end furthest from the predatory stimulus; in all trials, the eels freely explored the tank immediately on release. All eels received one of three treatments, consisting of two consecutive 2 min playback tracks from different harbours (i) an ambient-noise track followed by another ambient-noise track (control treatment), (ii) an ambient-noise track followed by a ship-noise track (additional-noise treatment), or (iii) a ship-noise track followed by an ambient-noise track (post-additional-noise treatment). Eels were randomly allocated in an independent-measures design to the three sound treatments. During playback of the second track, and directly after the eel entered the strike zone of the predator (a 20×20 cm section in front of the endpoint of the looming stimulus), the observer remotely released the predator. No significant difference was found between treatments in the time eels took to arrive at the strike zone (Kruskal–Wallis test: $\chi_{2}^{2}=1.23$, *p*=0.542). All trials were recorded with a HD video recorder (Casio EX-FH20, Tokyo, Japan).

The fish were measured (total length, range 71.5–160.0 mm) and weighed (scales Kern EG420-3NM, Kern & Sohn GmbH, Balingen, Germany; blotted wet mass, range 0.23–4.87 g) directly after the experiments. Body condition of the fish was calculated using Fulton’s condition factor. No significant differences between eels in the treatments were found with respect to size, mass or condition (Kruskal–Wallis tests, length: $\chi_{2}^{2}=0.11$, *p*=0.995; weight: $\chi_{2}^{2}=0.79$, *p*=0.961; ANOVA, condition: *F*_2_=0.29, *p*=0.746).

Eels were tested in 11 blocks of 18 individuals (six fish in each of the three treatments, one for each possible combination of playback tracks from the different harbours); missing data from three individuals made a total of 195 observations. Using a block design ensured balancing of treatment and playback tracks relative to time. Order of playback track was randomized within blocks, and subsequent analysis confirmed no bias in the ordering of the treatments (Kruskal–Wallis tests all *p*>0.313). Water in the experimental tank was changed between blocks; within blocks, the water was stirred to homogenize potential olfactory cues. To determine whether the eel startled in response to the looming stimulus, videos of each trial were analysed without sound; the scorer was therefore ‘blind’ to the treatment. Eels were recorded as startling if they exhibited a directional change in swimming trajectory between sequential frames. For those individuals that startled, the time taken to startle (from initiation of predator release) was also determined.

### 2.4 Eel ventilation experiment

We examined potential immediate and lasting impacts of additional noise on physiology by considering ventilation rate, measured as opercular beat rate (OBR). Increased ventilation rates are common indicators of greater stress levels [47] and high ventilation rates are positively related to high dissolved oxygen consumption in juvenile eels [26]. Individual eels were placed into an airtight 30 ml see-through polypropylene tube (wall thickness 1 mm with approx. 90–95% acoustic transparency), that was positioned 10 cm side-ways from a loudspeaker on the floor of a plastic test tank. The test tank measured 34×20 cm with a 16 cm water depth, had a 2 mm wall thickness and a side-ways facing underwater speaker at one end hidden behind an opaque polypropylene divider (thickness 3 mm).

Eels were randomly allocated in an independent-measures design to two treatments, both of which consisted of three consecutive 2-min playback tracks (i) an ambient-noise track (initial period), followed by a ship-noise track recorded at a different harbour (acute-noise period), and finally another ambient-noise track from the third harbour (recovery period) or (ii) three different ambient-noise tracks. In the predation experiment, the relevant fish response (startle to a looming stimulus) can be determined only once during a treatment (during one particular playback track), necessitating the use of three treatments to consider how current and recent noise exposure both affect anti-predator behaviour relative to a control condition. By contrast, OBR can be measured during each playback track in a given treatment, so only two treatments were needed in this experiment to consider the same question about the effect of current and recent noise exposure. Eels measured between 68 and 131 cm and weighed between 0.21 and 2.67 g. The eels allocated to the two treatments did not differ significantly in size, mass or condition (independent-samples *t*-tests, length: *t*_154_=−0.66, *p*=0.509; mass: *t*_154_=−0.66, *p*=0.512; condition: *t*_154_=−0.65, *p*=0.516). During each of the 2-min periods in a given trial, OBR was counted for 60 s. If opercular beats could not be observed, counting was paused and continued directly after the operculum was visible again (always less than 90 s for 60 s of counting). Activity levels were scored on a 3-point ordinal scale (0=no activity, 1=some activity, 2=very active; as in [48]); no significant differences in activity were found between the treatment groups in each playback period (Mann–Whitney *U*-tests: *n*_1_=*n*_2_=78; initial period: *Z*=−0.60, *p*=0.546; acute-noise period: *Z*=−0.48, *p*=0.631; recovery period: *Z*=−0.70, *p*=0.485).

One hundred and fifty-six eels were tested in 13 blocks of 12 individuals (six fish in each of two treatments, one for each possible combination of playback tracks, without reusing recordings taken at the same location per trial). Order of noise treatments and playback tracks were counterbalanced within blocks. After each trial, the tube was rinsed and refilled with fully aerated water.

### 2.5 Seabass

Farmed juvenile European seabass were sourced from Ifremer (Plouzané, France) and transported to the University of Exeter. After approximately two months, the fish were transported to the University of Bristol where they were held in two 450 l glass aggregation tanks for minimally two weeks to acclimatize. At least one week before the start of the experiment, fish were transferred to a 50 l glass holding tank located in the experimental room. All fish were held with constant temperature (18.0±1.0°C), in a 13 L:11 D regime, and were fed daily with ZM-300 food (zmsystems.co.uk). European seabass can detect sound pressure frequencies between 100 and 500 Hz [49], which coincide with the dominant frequencies of pile driving noise [40].

### 2.6 Seabass sound recordings and playbacks

**Figure 3.**
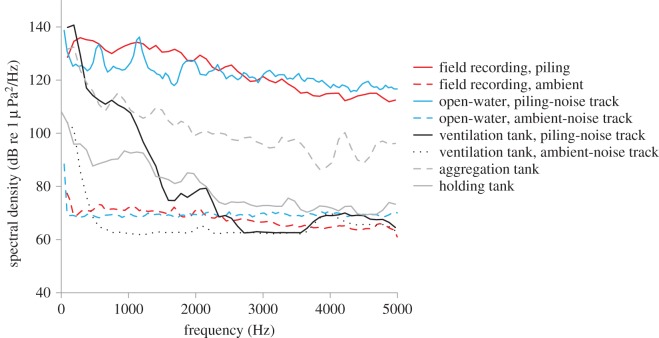
Spectral-level analyses of field and tank-based recordings for seabass. The figure includes baseline conditions in the aggregation and holding tanks, field recordings of ambient offshore noise and pile driving, ambient-noise and piling-noise playback in open-water conditions, and playback of ambient-noise and piling-noise playback tracks in the tank-based ventilation rate experiment (Ventilation tank). FFT analysis of sound 50–5000 Hz, using Avisoft SASLabPro v. 5.2.07: spectrum-level units normalized to 1 Hz bandwidth, Hann evaluation window, 50% overlap, FFT size 1024, averaged from a 1 s sample of each recording (one pile strike per sample), 43 Hz intervals are presented.

Baseline captive noise conditions were established 2 cm above the tank bottom at the side of the large aggregation tanks—where the seabass were most commonly found—and in the centre of the holding tanks (see figure 3 for acoustic conditions). Acoustic recordings for the laboratory-based ventilation experiment were made 10 cm from the speaker just above the tank floor at the location where the bass were tested. In the open-water experiment (see details further on), sound recordings were made 1 m from the speaker at the position the fish were tested.

We used the same recordings of ambient harbour noise as in the eel experiments (see before). Pile-driving recordings were made in Swansea Bay, UK of a 1.2 m diameter monopole driven *ca* 25 m into the seabed with a 6.5 m water depth. Recording were made between 87 and 200 m from the pile using a Hi Tech Inc. HTI-99HF omnidirectional hydrophone (with inbuilt preamplifier, manufacturer calibrated sensitivity −204 dB re 1 V/μPa, 0.02–125 kHz frequency range) and an EASDA recorder (Rtsys, 44.1 kHz sampling rate, Brittany, France; see figure 3 for an example of the recordings). Recordings were cut into 2-min segments using Audacity 1.3.13 (as for eel experiments), to create three different ambient-noise and piling-noise playback tracks. Sound levels of the tracks of each type were modified to play at approximately equal pressure levels, when played back using an underwater speaker; both laboratory and open-water experiments used the same recordings. Acoustic propagation in small tanks consists of more complex propagation and reverberation than acoustics in open-water conditions [41]; this makes confirmation of findings from tanks in open-water conditions important. The laboratory experiment used a laptop (Toshiba Satellite Pro A300), amplifier (Phonic max 500) and an underwater speaker (Aqua30, DNH; effective frequency range 0.08–20 kHz; www.dnh.no) to playback the noise, and the open-water experiment used a Philips GoGear VIBE WAV/MP3 player (Amsterdam, The Netherlands), an M033N amplifier (Kemo Electronics GmbH; 18 W; frequency range *ca* 0.04–20 kHz) and a UW-30 underwater speaker (as before), powered by a battery (12 V, 7.2 Ah sealed lead-acid). The three ambient-noise playbacks in the centre of the ventilation tank (where the fish were tested) measured between 103.1 and 105.0 dB RMS re 1 μPa, whereas ambient-noise playbacks in the open-water experiment (see below) measured between 66.8 and 67.2 dB RMS re 1 μPa; all broadband RMS values were calculated over 1 min, frequency range 50–5000 Hz. In tanks, low-frequency acoustic background levels (approx. 10–500 Hz) can be relatively high due to noises generated by oxygenation, water filters and common building noise. Moreover, differences in sound propagation (i.e. more reverberation and reflection in tanks compared with open-water conditions) are likely to account for acoustic differences. Because impulsive sounds are not well represented by RMS values [50], peak value, 90% energy pulse duration and pulse frequencies (pulses min^−1^) are reported. In the ventilation tank during additional noise, the peak value of an example pulse per playback measured 165.5, 166.0 and 167.3 dB re 1 μPa with a 392.1, 456.7 and 584.0 ms 90% energy pulse duration; pulse frequencies were 39, 32 and 40 pulses min^−1^. In the open-water experiments during piling-noise playbacks, the peak values of an example pulse per playback measured 200.1, 200.7 and 201.5 dB re 1 μPa with a 206.3, 203.9 and 249.5 ms 90% energy pulse duration; pulse frequencies were as above.

### 2.7 Seabass ventilation experiments

Seabass ventilation experiments (performed in both laboratory and open-water conditions) followed the same general protocol as for the eel ventilation experiment. Seabass received one of two sound treatments (additional-noise treatment and control treatment) involving three consecutive 2-min periods that were respectively (i) an ambient-noise track (initial period), followed by a piling-noise track (acute-noise period), and finally a different ambient-noise track from the initial period (recovery period) or (ii) three different ambient-noise tracks. To reduce pseudo-replication, 12 playback combinations were made. During each 2-min period, OBR was counted for 60 s (always less than 90 s required to obtain 60 s counting). No significant treatment differences in ventilation rates between initial periods were found in the laboratory and open-water experiments (independent-samples *t*-test: *t*_154_=1.55, *p*=0.124).

#### 2.7.1 Laboratory

Individual seabass were placed into a cylindrical-shaped polystyrene mesh tube (9×3 cm, mesh size 2 mm) and positioned 10 cm from a loudspeaker (side-ways facing and hidden behind an opaque polystyrene divider; thickness 3 mm) on the floor of a 55 l glass aquarium. Twenty seabass (measuring 36.0–50.0 mm standard length (SL), weighing 0.68–2.15 g) were tested in each sound treatment, with order of treatment and playback tracks counterbalanced. No significant treatment differences were found between the seabass in size, mass or condition (independent-samples *t*-tests, length *t*_38_=−1.41, *p*=0.168; mass: *t*_38_=−1.25, *p*=0.218; condition: *t*_38_=0.49, *p*=0.628). Activity levels were scored on the same 0–2 scale used in the eel experiment; no significant differences in activity levels were found between treatment groups per playback period (Mann–Whitney *U*-tests: *n*_1_=*n*_2_=20; initial period: *Z*=−0.64, *p*=0.524; acute-noise period: *Z*=−0.74, *p*=0.457; recovery period: *Z*=−1.68, *p*=0.157).

#### 2.7.2 Open water

The open-water experiment was conducted in Bristol Harbour (Cumberland Basin, Bristol, UK) using 36 seabass (measuring 42–64 mm SL), with 18 fish in each sound treatment tested in counterbalanced order. Fish were transferred to the harbour by car (less than 10 min) and kept in 80 l containers provided with fresh oxygen. In each trial, one fish was transferred from a holding container to a glass jar (15 cm length, 9 cm diameter, 4 mm thickness; the jar was acoustically transparent for low-frequency wavelengths (wavelengths<jar diameter; approx. 17 kHz) and does not impede or trap sound) that was attached to a pole with rubber bands. The pole was equipped with a camera (GoPro, Hero3; positioned 25 cm from the jar) to record the fish during the playback trials. The set-up (the pole including jar and camera) was then lowered 80 cm under the water surface (water depth harbour 4 m) at 1 m from the speaker (also located 80 cm under the water surface). The playback treatment started directly after the set-up was lowered to the correct position. After each trial, the jar was rinsed and filled with aerated water for the next trial. To eliminate observer bias, all videos were analysed without sound. Activity levels were not measured due to logistical problems.

### 2.8 Statistics

Anti-predator behaviour (startle, no startle) was compared between sound treatments with a *χ*^2^ test; startle latency between all treatments was tested with a Kruskal–Wallis test and with Mann–Whitney *U*-tests to analyse between individual treatments. Ventilation rates were tested using repeated-measures ANOVAs, with sound treatment, period and their interaction included as predictor variables. Changes in ventilation rates per period were tested with independent-sample *t*-tests if the data were normally distributed or with Mann–Whitney *U*-tests if not. No significant differences were found in any response variable between playback tracks of the same sound treatment (all *p*-values>0.432). All statistical testing was conducted on absolute values in IBM SPSS Statistics (v. 20.0.0).

## 3. Results

### 3.1 Eel experiments

**Figure 4.**
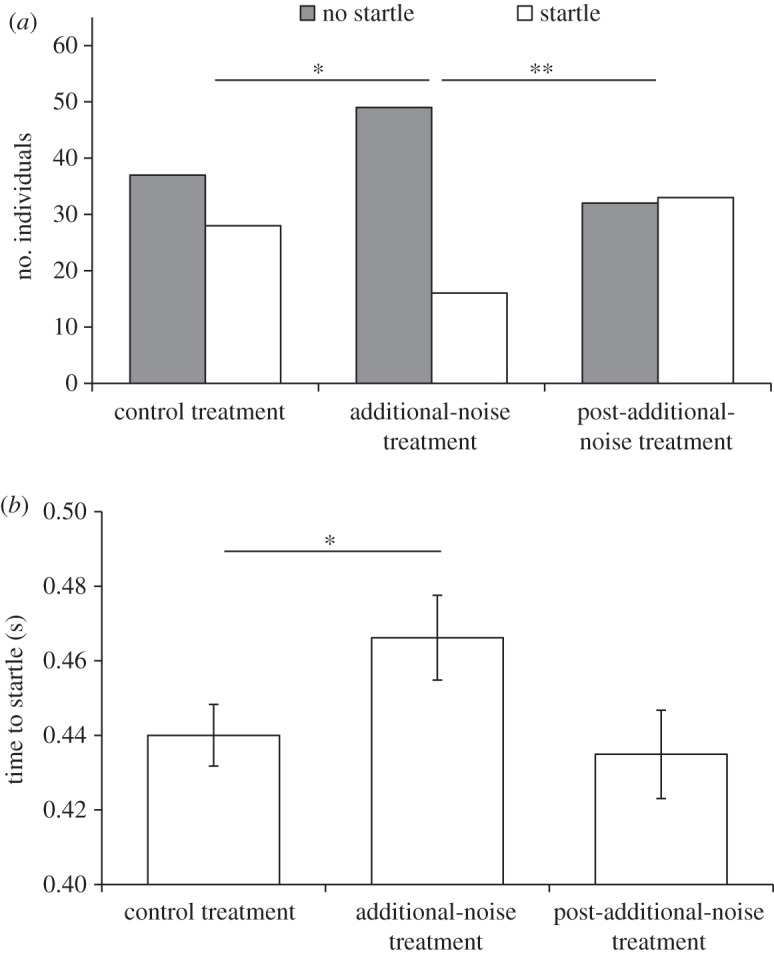
Eel anti-predator responses during ambient-noise playback following another ambient-noise playback (control treatment), ship-noise playback following ambient-noise playback (additional-noise treatment) and ambient-noise playback following ship-noise playback (post-additional noise treatment). (*a*) Number of individuals exhibiting a startle response or not to a looming stimulus (*n*=65 per treatment). (*b*) Time taken to startle to the looming stimulus for the eels that exhibited a startle response; shown are mean±s.e. Control treatment *n*=38, additional-noise treatment: *n*=16, post-additional noise treatment: *n*=33. Asterisks (* and **) denote *p*<0.05 and *p*<0.01, respectively.

Playback treatment significantly affected the likelihood of eels startling to the looming predatory stimulus (*χ*^2^ test $\chi_{2}^{2}=0.22$, *n*=195, *p*=0.007; figure 4*a*). Eels experiencing a ship-noise playback track when the stimulus was released were 42.9% less likely to startle than individuals in the control treatment ($\chi_{1}^{2}=0.19$, *n*=130, *p*=0.026). Eels in the post-additional-noise treatment (those that had recently experienced ship-noise playback but were exposed to an ambient-noise track at the time of stimulus release) were 51.5% more likely to startle to the looming stimulus than those in the additional-noise treatment ($\chi_{1}^{2}=0.26$, *n*=130, *p*=0.002; figure 4*a*). Eels in the post-additional-noise treatment did not therefore differ significantly from control-treatment individuals in their likelihood of startling ($\chi_{1}^{2}=0.08$, *n*=130, *p*=0.380; figure 4*a*), suggesting complete recovery.

There was no overall significant difference between sound treatments for the time taken to startle to the looming stimulus (Kruskal–Wallis test $\chi_{2}^{2}=4.27$, *n*=77, *p*=0.118; figure 4*b*). However, eels that startled during ship-noise playback were significantly slower to react (6.0%) than those that startled in the control treatment (Mann–Whitney test: *Z*=−2.20, *n*_1_=28, *n*_2_=16, *p*=0.028; figure 4*b*). Startle response times of eels in the post-additional-noise and additional-noise treatments did not differ significantly, although the latter individuals were 7.2% slower to react (*Z*=−1.52, *n*_1_=16, *n*_2_=33, *p*=0.129; figure 4*b*). Eels in the post-additional-noise treatment were not significantly different in their startle response times to those in the control treatment (*Z*=−0.38, *n*_1_=28, *n*_2_=33, *p*=0.705), suggesting rapid recovery following acoustic disturbance.

**Figure 5.**
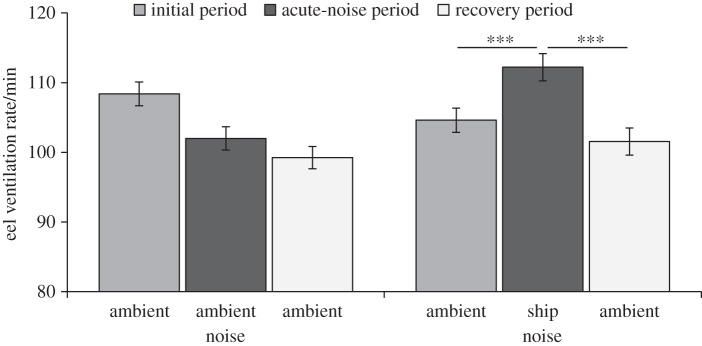
Mean±s.e. ventilation rates of eels during three consecutive 2-min periods (initial period, acute-noise period, recovery period) of either different ambient-noise playbacks (control treatment) or an ambient-noise playback followed by ship-noise playback followed by ambient-noise playback (additional-noise treatment). *N*=78 per treatment. Asterisks (***) denote *p*<0.001.

Eel ventilation rate was significantly affected by the interaction between playback treatment and trial period (repeated-measures ANOVA treatment×period: *F*_2,153_=92.66, *p*<0.001; treatment: *F*_1,154_=1.47, *p*=0.227; period: *F*_2,153_=65.56, *p*<0.001; figure 5). While eels experiencing consecutive ambient-noise tracks exhibited a 5.8% decrease in ventilation rate between the first two periods (indicating continued lowering of stress levels potentially caused by capture), individuals that were switched to a ship-noise track showed a 7.4% increase in ventilation rate (indicating higher stress levels); there was a significant difference between treatments in the change in ventilation rate from initial to acute-noise periods (Mann–Whitney test: *Z*=9.53, *n*=156, *p*<0.001). Once the ship-noise track was switched to another ambient-noise track, eels in that additional-noise sound treatment immediately exhibited a decrease in ventilation rate (9.5%) compared with previous track, which was significantly greater in magnitude than the continued decrease in ventilation rate shown by control-treatment eels (2.4%; *Z*=6.17, *n*=156, *p*<0.001). However, the difference in ventilation rate between initial and recovery periods was significantly greater in the control treatment (8.1%) than in the additional-noise treatment (2.8%; *Z*=4.09, *n*=156, *p*<0.001), suggesting incomplete recovery following ship-noise exposure.

### 3.2 Seabass experiments

**Figure 6.**
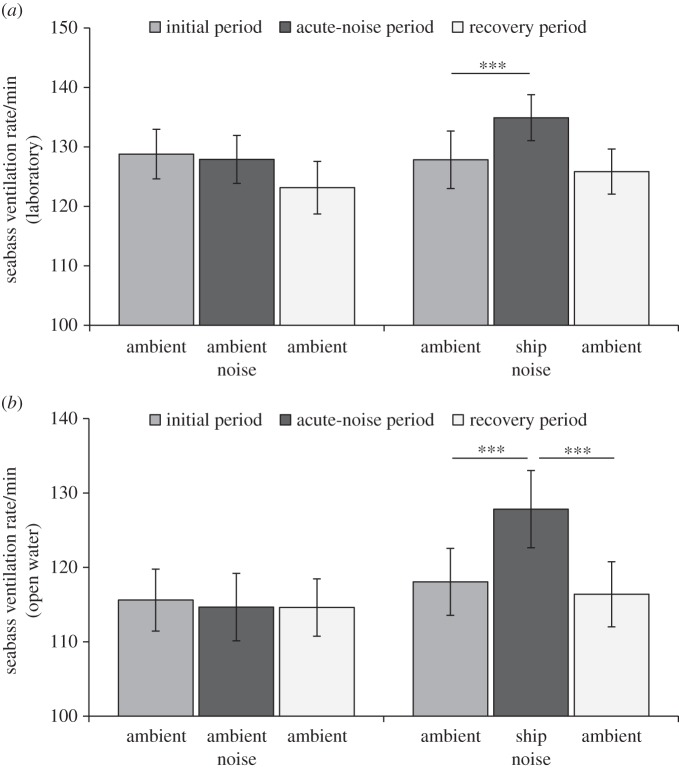
Mean±s.e. ventilation rates of seabass in a laboratory (*a*) and open-water (*b*) set-up during three consecutive 2-min periods (initial period, acute-noise period and recovery period) of either different ambient-noise playbacks (control treatment) or an ambient-noise playback followed by pile-driving-noise playback followed by ambient-noise playback (additional-noise treatment). *N*=20 per treatment in the laboratory experiment; *N*=18 per treatment in the open-water experiment. Asterisks (***) denote *p*<0.001.

In the laboratory experiment, seabass ventilation rate was significantly affected by the interaction between sound treatment and trial period (repeated-measures ANOVA treatment×period: *F*_2,38_=3.59, *p*=0.032; treatment: *F*_1,39_=0.26, *p*=0.611; period: *F*_2,38_=10.83, *p*<0.001; figure 6*a*). Seabass experiencing successive ambient-noise tracks showed a 0.6% decrease in ventilation rate between the first two periods, whereas individuals that were switched to a piling-noise track exhibited a 6.5% increase in ventilation rate; there was a significant treatment difference in the change in ventilation rate between the initial and acute-noise periods (independent-samples *t*-test: *t*_38_=−2.97, *p*=0.005). Seabass exhibited a decrease in ventilation rate between acute-noise and recovery periods in both control (3.8%) and additional-noise (6.5%) treatments; there was no significant difference between treatments in this change (*t*_38_=1.52, *p*=0.138). Since individuals in both treatments exhibited a non-significant difference in ventilation rate between initial and recovery periods (control treatment: 4.4%; additional-noise treatment: 0.7%; *t*_38_=−1.09, *p*=0.283; figure 6*a*), recovery seems complete in the immediate aftermath of the piling-noise playback.

As in the laboratory experiment, ventilation rate of seabass in the open-water experiment was significantly affected by the interaction between playback treatment and trial period (repeated-measures ANOVA treatment×period: *F*_2,34_=24.36, *p*<0.001; treatment: *F*_1,34_=0.87, *p*=0.357; period: *F*_2,34_=21.60, *p*<0.001; figure 6*b*). Seabass experiencing successive ambient-noise tracks showed a 1.0% decrease in ventilation rate between the first two periods, whereas individuals that were switched to a piling-noise track exhibited an 8.2% increase ventilation rate; there was a significant treatment difference in the change in ventilation rate between initial and acute-noise periods (independent-samples *t*-test: *t*_34_=−5.68, *p*<0.001). There was also a significant difference between treatments in the change in ventilation rate from acute-noise period to recovery period (control treatment: 0.5% decrease; additional-noise treatment: 8.6% decrease; *t*_34_=5.62, *p*<0.001). The lack of a significant treatment difference in the change in ventilation rate between initial and recovery periods (control treatment: 0.6%; additional-noise treatment: 1.3%; *t*_34_=0.43, *p*=0.670; figure 6*b*) suggests complete recovery in the immediate aftermath of piling-noise playback.

## 4. Discussion

Our experiments provide confirmation that short-term exposure to additional noise (playback of recordings of ship noise) affects both anti-predator startle behaviour and ventilation rate in juvenile eels [26], and demonstrate for the first time that the ventilation rate of juvenile seabass is affected in a similar way (to playback of recordings of pile driving). Our experiments on seabass indicate that this is not simply an artefact of conducting noise experiments in small tanks, since qualitatively similar findings were obtained in both the laboratory and open-water conditions, even though acoustic conditions were different. Crucially, we offer strong evidence that these two commercially important fish species can show rapid recovery following acute short-term exposure once such acoustic disturbance ceases, at least with respect to the measured behavioural and physiological responses and following the sound-exposure levels in these experiments. In the immediate aftermath of short-term noise exposure (the first 2 min following the 2-min noise exposure), both species showed extensive recovery to control levels; complete recovery in the case of eel anti-predator startle responses and seabass ventilation rate; complete recovery for eel startle latency and partial recovery in the case of eel ventilation rate. The consistency between species in response measures to additional-noise sources, both in initial impacts and subsequent rapid recovery, is important but future studies in natural conditions with real-world noise sources are needed moving forward.

If an anti-predator startle response, which indicates predator detection and is an essential part of a typical defence cascade [51], becomes less likely or slowed during and/or following additional noise, then prey would potentially be more vulnerable (depending on how the predators are themselves affected by noise). However, if recovery from short-term noise exposure is rapid, then the fitness consequences may be lessened; survival may only be compromised during actual periods of noise pollution. In the case of other behaviours previously shown to be affected by additional noise (e.g. decreased foraging and increased within-group aggression [8,24]), temporary effects of short-term exposure may have little lasting influence if there is an opportunity to compensate in quieter periods, especially if there is rapid recovery from any initial impact. Similarly, if any stress arising from short-term additional-noise exposure is short-lived (as indicated by our results), lasting impacts on growth and survival may be less likely. However, if noise exposure causes physiological damage, e.g. hearing threshold shifts [34,35], injury or death [12,21], then recovery or compensation during less noisy periods might be difficult or even impossible. Considering the impact of longer and repeated exposure of noise on aquatic organisms is important and has consequently been stressed by several reviews [6,52]. In fishes, impacts of repeated and chronic noise exposure on development and growth have shown mixed results, with some studies finding adverse reactions (e.g. [53,54]), while others did not find impacts [55–57]. Ultimately, the noise intensity, the animals’ sensitivity to noise and the temporal regime of acoustic events (e.g. a single daily ferry versus busy shipping lanes and harbours) will influence the likelihood and speed of recovery and the timeframe over which animals may compensate for noise-pollution events.

Since both European eels and seabass are likely to encounter different situations regularly during (seasonal) migrations, they might be particularly good at adjusting rapidly to changing acoustic conditions; this remains to be tested. In general, migratory species may be less flexible during non-migratory life stages, when it may pay to be adapted to standard local conditions. Non-migratory species may not necessarily have the option to move away from a noise source, and might therefore be even more vulnerable to anthropogenic disturbance if they recover less quickly from a given noise exposure. Thus, while most work examining the impact of anthropogenic noise has considered a single species, studies comparing the responses of different species are important [58–60]. That is partly because interspecific differences in susceptibility are likely due to, for example, variation in hearing ability [61] and different mechanisms of physiological stress response [62], but also because there may be strategic life-history related differences in their responses to environmental change.

Compared to a previous study on eels using the same general looming-predatory stimulus set-up [26], we found a much lower startle rate even in the control treatment. This could be due to some small, unknown difference in the lighting or other conditions, making the stimulus less easy to detect, or it could result from difference in the prior experiences of the two eel cohorts; previous acoustic experiences have been shown to influence, for example, orientation and alternative mating strategies [63,64]. Additionally, eel ventilation rates in the control treatment in this study decreased from the initial to the next period, whereas this was not found in Simpson *et al.* [26]. One possibility for this difference might be that, for some reason, eels in the current study were more stressed by their capture and placement in sealed tubes and thus took longer to recover to a baseline ventilation rate. Whatever the reasons for these differences between studies, current work confirms the main results found before—that there is a significant difference in behaviour and physiology depending on noise treatment, with additional noise having a potentially detrimental effect during the period of exposure.

Unlike other pollutants, such as ocean acidification and toxin accumulation [2,3], acoustic noise quickly dissipates if the source is turned off and thus transitory effects are plausible. Our results offer potential optimism regarding the possibility of rapid recovery following short-term acoustic disturbance on behaviour (anti-predator startle response) and physiology (ventilation rate). However, there is a cautionary note there were significant initial effects of additional noise on both response measures, and the animals may still carry some costs of that experience, or accrue other costs not measured in this study, even if they have returned to levels equivalent to control individuals in certain regards. Future work needs to consider the responses of animals in natural conditions to real-world noise sources, and to longer or repeated exposures where residual effects may be more likely, since understanding both the immediate and lasting effects of noise is crucial for the development of mitigation measures and regulatory decisions.

## Data accessibility

The datasets supporting this article can be found in the Dryad digital repository http://dx.doi.org/10.5061/dryad.7b0v8.
